# Matrix Dynamics and Microbiome Crosstalk: Matrix Metalloproteinases as Key Players in Disease and Therapy

**DOI:** 10.3390/ijms26083621

**Published:** 2025-04-11

**Authors:** Paraskevi Ioannou, Elias Katsoulieris, Nikolaos A. Afratis

**Affiliations:** 1 Laboratory of Biotechnology and Molecular Analysis, Department of Agricultural Development, Agri-Food & Management of Natural Resources, National and Kapodistrian University of Athens, Evripos Campus, 34400 Psachna, Evia, Greeceelkatsou@agro.uoa.gr (E.K.); 2 Department of Immunology and Regenerative Biology, Weizmann Institute of Science, 234 Herzl Street, Rehovot 7610001, Israel

**Keywords:** microbiome, matrix metalloproteinases, matrixbiome, extracellular matrix

## Abstract

Matrix metalloproteinases (MMPs) are key enzymes involved in extracellular matrix (ECM) remodeling, regulating a wide range of cellular and immune processes in both homeostatic and pathological conditions. Host–microbiota interactions play a critical role in maintaining ECM balance; however, during dysbiosis, this regulation is disrupted, leading to compromised barrier integrity, pathogen translocation into circulation, and the development of systemic diseases and cancer. This review highlights the bidirectional relationship between MMP expression/activity and microbiota dysbiosis, emphasizing tissue-specific alterations in MMP activity that contribute to disease progression. In addition, it integrates interdisciplinary evidence to illustrate the MMP-dependent mechanisms underlying various pathologies associated with oral and gut microbiome dysbiosis, including long-range effects through the gut–skin and gut–brain axes. Thus, this review introduces the emerging field of *MatrixBiome*, which explores the complex interactions between the ECM, microbiota, and host tissues. Finally, it also outlines therapeutic strategies to modulate MMP levels, either indirectly through microbiome-targeted approaches (*e.g.*, prebiotics, probiotics, and postbiotics) or directly using MMP inhibitors, offering promising avenues for future clinical interventions.

## 1. Introduction

The human microbiota is a highly dynamic and complex ecosystem composed of a diverse range of microorganisms, including bacteria, archaea, viruses, bacteriophages (phages), and fungi, each contributing uniquely to host health and disease.

Eubiosis and dysbiosis are fundamental concepts in understanding the role of human microbiota in health and disease [1]. Eubiosis refers to a state of microbial balance in which the symbiotic relationship between the host and its microbiota supports essential physiological functions, including nutrient absorption, immune regulation, and defense against pathogens [1,2]. In contrast, dysbiosis describes a disrupted microbial ecosystem marked by decreased diversity of beneficial species and an overgrowth of pathogenic species, often leading to adverse health outcomes [3]. This imbalance has been associated with various conditions, including inflammatory bowel disease (IBD), obesity, colorectal cancer, etc. [4] Several factors can contribute to the transition from eubiosis to dysbiosis, including dietary imbalances, antibiotic use, infections, and chronic stress [1]. Understanding the mechanisms driving these shifts is essential for developing interventions aimed at restoring microbial homeostasis and promoting overall health.

Microbial communities colonize various body sites, such as the gastrointestinal tract (gut), oral cavity, skin, and respiratory system (both upper and lower airways) [3]. The gut microbiome is particularly abundant and diverse, harboring trillions of microbes that contribute to nutrient metabolism, immune system development, and the production of bioactive compounds such as short-chain fatty acids (SCFAs) and essential vitamins (*e.g.*, B vitamins and vitamin K) [5]. In the oral cavity, microbial communities contribute to oral health, but can also promote pathologies, such as dental caries, periodontal diseases, cancer, and neurodegenerative diseases, when disrupted [6]. The skin microbiome plays a crucial role in forming a protective barrier against pathogens and supporting wound healing, while the respiratory microbiome is essential for immune defense and mucosal integrity [7,8]. Together, these microbiomes form a complex network of host–microbe interactions essential for maintaining homeostasis and protecting against various diseases. In recent years, the human microbiome has emerged as a significant player in cancer progression. As highlighted by Hanahan et al. in the context of the Hallmarks of Cancer, microbial communities influence tumorigenesis by modulating inflammation, shaping immune responses, and facilitating interactions between microbial metabolites and cancer cells [9]. 

Extracellular matrix (ECM) is a highly dynamic and intricate structure composed of key components, such as collagen, proteoglycans, glycosaminoglycans, elastin, elastic fibers, laminins, and fibronectin [10]. Under development, repair, and regeneration conditions, ECM is continuously remodeled to maintain tissue homeostasis. However, disruptions in this finely regulated balance can contribute to disease progression and pathogenesis [10,11]. ECM proteases play a key role in regulating the turnover of ECM proteins. These degrading enzymes, which belong to the metzincin family, include matrix metalloproteinases (MMPs), disintegrin and metalloproteinases (ADAMs), and disintegrin and metalloproteinases with thrombospondin motifs (ADAMTSs). Among these proteases, MMPs are the most prominent, and their expression levels are significantly altered in pathological conditions, leading to excessive ECM proteolysis [12,13].

The interaction of ECM with microbiota regulates human homeostasis, however during microbial dysbiosis, invasive microorganisms damage epithelial barriers, leading to pathological conditions. Here, we introduce MatrixBiome, a scientific field that aims to specifically explore the interaction of microbiome with ECM and extend these findings to both physiological and pathological conditions. Given that the ECM is a complex network composed of various macromolecules that regulate homeostatic and pathological mechanisms through mechanical, signaling, and inflammatory pathways, we focus on a sub-field of MatrixBiome that examines the specific interactions between MMPs, microbiota, and host tissues. This interplay is fundamental to the regulation of various biological mechanisms across different tissues, and its comprehensive understanding may facilitate the development of novel therapeutic strategies for diverse pathological conditions, including inflammatory diseases and cancer. In the following sections, the impact of MMP-mediated proteolysis in MatrixBiome crosstalk is elucidated. We prompt future investigations into every relevant disease pathway to consider MatrixBiome components and processes, and suggest future collection of relevant material for reviews that will fill all the gaps regarding MatrixBiome relevance to human disease.

## 2. Matrix Metalloproteinases (MMPs)

### 2.1. Structure, Function, and Regulatory Mechanisms of MMPs

**Figure 1:**
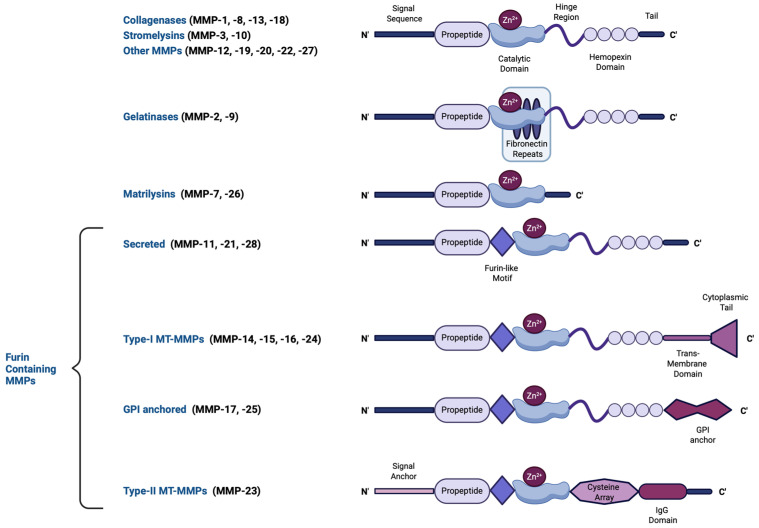
A schematic representation of the major MMP subtypes and their respective structural domains. MMPs share a common core structure consisting of a propeptide, a catalytic metalloproteinase domain, a linker peptide (hinge region), and a hemopexin domain. The propeptide contains a cysteine switch motif (PRCGXPD) that binds the catalytic Zn^2+^ ion, keeping the MMP in its latent pro-MMP form. The catalytic domain features the Zn^2+^-binding motif (HEXXHXXGXXH). Structural variations define different MMP subtypes: gelatinases (MMP-2, -9) have three type-II fibronectin repeats within the catalytic domain, matrilysins (MMP-7, -26) lack both a hinge region and a hemopexin domain, and furin-containing MMPs (*e.g.*, MMP-11, -21, -28) possess a furin-like recognition sequence for intracellular activation. Membrane-type MMPs (MT-MMPs) contain transmembrane or glycosylphosphatidylinositol (GPI) anchors, while MMP-23 features a unique cysteine-rich region and an immunoglobulin-like proline-rich domain. This structural diversity influences MMP function, activation mechanisms, and substrate specificity. This figure was created with BioRender.com.

The human MMP family contains 23 human endopeptidases characterized by their zinc-dependent catalytic activity. These enzymes play essential roles in degrading ECM components, including collagen, elastin, and proteoglycans, enabling tissue remodeling and repair. MMPs can be either intracellular (including mitochondrial and nuclear), secreted, or membrane-bound [14,15]. The structure of most MMPs comprises a propeptide, a catalytic metalloproteinase region, a hinge region, and a hemopexin-like (linker; heme-binding) domain [16], the latter acting as interacting regions with tissue inhibitors of metalloproteinases (TIMPs), the endogenous MMP inhibitors, and also conferring additional substate specificity, as well as providing protease recognition motifs that serve as activation sites [17]. A few MMPs, however, including matrilysins, are of the so-called minimal domain MMPs, meaning that they lack the linker domain and thus, possess a broader range of targets with which they bind with reduced specificity [18]. MMPs have been classified—based on the substrate they cleave, their localization, and their source—into collagenases (MMP-1, MMP-8, MMP-13, and MMP-18), gelatinases (MMP-2 and MMP-9), stromelysins (MMP-3, MMP-10, and MMP-11), matrilysins (MMP-7 and MMP-26), membrane-type MMPs (MT-MMPs), and macrophage elastase (MMP-12) [19]. The structural diversity of MMPs, including variations in their propeptide, catalytic domains, and activation mechanisms, is illustrated in Figure 1. In addition, MMPs can cleave a variety of non-ECM components, including growth factors, adhesion receptors, and cytokines [19]. The ratio between MMPs and their inhibitors is a crucial parameter determining ECM turnover rates [20,21].

MMPs are initially synthesized as inactive pro-MMP enzymes, known as zymogens. To exert any function, MMPs must first be activated. In most cases, MMP activation requires cleavage of the propeptide, leading to the release of the active form of the enzyme. The role of the propeptide is to prevent the binding and cleavage of the substrate. This mechanism is mediated via the link of the sulfhydryl group of cysteine from the propeptide-conserved region (PRCGXPD) with an atom of Zn^+^ in the catalytic domain [16]. The cysteine–Zn^2+^ complex maintains MMPs in their inactive form. However, cross-reactions with other MMPs and proteases can disrupt this interaction, triggering the “cysteine switch” mechanism, which subsequently leads to MMP activation [22]. Additionally, multiple other factors account for the induction of MMP activity, including MMP compartmentalization, dimerization, reactive oxygen/nitrogen species (*e.g.*, peroxynitrite), allostery following interaction with substrates, interaction with integrins and cell surface receptors, or via interaction with other MMP members [23]. Although most secreted MMPs become activated once released from the cell of origin, a few undergo intracellular activation which relies on post-translational modifications, such as phosphorylation, that induce an active, fully structurally retained zymogen [19]. This significantly impacts current MMP inhibitors designed based on the cleaved MMP sequence [14]. Membrane-bound MMPs (*e.g.*, MT-MMP) are activated following cleavage of their furin-recognition domains, contained within the propeptide, by furin proteinases [16]. Following MMP activation, their proteolytic activity is regulated by their endogenous inhibitors, TIMPs [24]. An imbalance between MMP activation and TIMP-induced inhibition can contribute to either the progression or suppression of various diseases [20]. Nevertheless, TIMP-induced regulation of MMP activity is complex and does not necessarily imply an inhibitory effect over a given MMP; rather it involves intermediate interactions of TIMPs with alternate MMP members or propeptides to sufficiently promote either activation or inhibition of that given MMP [25,26]. In humans, TIMPs are categorized into a family of four members: TIMP-1, TIMP-2, TIMP-3, and TIMP-4. Their expression is generally tissue-specific, except for TIMP-2, which is ubiquitously expressed throughout the body [20].

### 2.2. The Role of MMPs in ECM Remodeling

The ECM turnover is a constitutive process that applies to all tissues and drives all responses to environmental and homeostatic stimuli, including signaling between ECM components and surrounding tissue, cellular differentiation, and migration. It involves modifications in the abundance, structure, and organization of over 700 proteins that altogether define the net 3D structure of the ECM and its biochemical and biophysical properties [27]. These changes encompass alterations of ECM component synthesis, post-translational modifications, degradation, and the integrin-binding-inducing force [28].

The most essential regulatory functions of MMPs in ECM turnover are exerted in the steps of synthesis, degradation, and signaling between ECM components and resident cells [19]. The latter largely involves intermediate signaling between growth factors or cytokines that are embedded within the ECM and amend liberation following MMP-mediated cleavage of their ECM protein anchors [28]. MMP-mediated ECM component synthesis involves complex counterbalances between TGF-β activation and inactivation states, all being MMP-mediated. In addition, cells utilize ECM component–MMP transcriptional loops to regulate ECM gene transcription according to the pericellular MMP activity [29]. For example, MMP-14-mediated collagen degradation results in the liberation of the previously collagen-occupied β1-integrins, which become activated to signal for MMP-14 transcriptional inhibition and subsequent native collagen deposition [29]. ECM breakdown greatly involves collagen fiber degradation, which is a three-step process, initiated by MMP binding to collagen. The second step involves the triple helicase activity of MMPs, that is, the unwinding of the three collagen helices so the scissile bonds can be accessible during the third step of catalysis, where they are cleaved. Additional sites termed exosites, located either on the linker domains or on the fibronectin II modules, provide additional specificity of collagenases and gelatinases to both denatured and native collagen, as well as to integrins [30]. However, the normal turnover of connective tissue matrices relies almost exclusively on the phagocytosis-mediated degradation of collagenous material by cathepsins, and much less by the functioning of MMP members, although it is generally accepted that all members can cleave ECM constituents [31,32].

As cleaved ECM substrate fragments can stimulate MMP activity, it appears that MMPs function through ECM remodeling to regulate various cellular processes, such as differentiation, migration, and immune interactions [33]. For instance, such a mode of MMP activation has been linked to the cleavage of laminins and of adherens junctions components, such as E- and VE-cadherins, as well as of integrins, overall initiating the cellular migratory process [29]. Furthermore, in many cases, multiple MMP types are working in parallel while orchestrating the proteolytical process. This multi-member MMP interplay is a common mechanism of regulation of normal processes but is also involved in pathological states. For example, mesenchymal stem cell differentiation involves an interplay between MT-MMPs, collagenases, and gelatinases, whereas an MMP member cleaves integrin receptors to initiate mitogen-activated protein kinase (MAPK) signaling that stimulates the expression of an alternate member, as well as collagen fiber degradation/de novo synthesis [34,35]. Similarly, in the cellular migratory process, MT-MMPs function as lead activators of MMP-2 or by shedding adhesion molecules [36]. MMP-induced angiogenesis is initiated by MMP-2 mediated basement membrane degradation and endothelial cell–cell adhesion cleavage to promote endothelial cell migration [37]. Additional MMPs catalyze growth factor (*e.g.*, VEGF) cleavage and subsequent activation/mobilization/liberation from the ECM, as well as the degradation of angiogenic inhibitors [38]. These active growth factors stimulate endothelial cells to secrete more gelatinases to enhance the angiogenic process [39]. Matrilysins, which show a broad target spectrum, are also participating in the process [40]. As all MMP-mediated processes are reciprocally regulated by alternate MMP members or cleaved fragments of substrate material, angiogenesis as well can be inhibited via the release of anti-angiogenic products following MMP-mediated collagen IV or plasminogen degradation [41]. Therefore, MMP activity is additionally regulated by catalytic product feedback mechanisms.

MMPs are crucial regulators of the innate immune response, and their effects are closely linked to microbiota dysbiosis. They participate in both inflammatory and anti-inflammatory responses and regulate chemotactic gradients, mainly by truncating chemokines and their receptors, or mobilizing them via cleavage of their proteoglycan anchors that render them within the ECM, thus providing selection over the immune cell types that regulate the inflammatory response [42,43]. In addition to their direct participation as inducers of signaling cascades that lead to cytokine generation, MMP-mediated ECM cleavage intermediate products, termed as matrikines, also participate in cytokine generation and chemotaxis [44,45]. MMP-8 is the main MMP driving PMN recruitment via activating cleavage of the chemokine CXCL8, while the macrophage-specific MMP12 cleaves and inactivates CXCL members, resulting in the suppression of PMN-mediated responses [45,46]. Gelatinases are mainly secreted by macrophages and neutrophils to facilitate basement collagen IV cleavage and cellular migration, as well as proinflammatory cytokine activation, while stromelysins enhance vascular permeability through basement membrane degradation and endothelial tight junction cleavage to facilitate immune cell infiltration to inflamed areas [47].

From all the above, it is apparent that MMP functions are not limited to ECM turnover, but their ECM-modulatory effects define a broad spectrum of biological processes. In the context of microbiomes, MMPs influence barrier integrity, immune signaling, and microbial colonization. For instance, MMP-mediated degradation of tight junction proteins in the gut epithelium facilitates microbial translocation, exacerbating conditions like IBD [19]. Similarly, in periodontitis, excessive MMP activity leads to connective tissue and alveolar bone destruction, driven by interactions with pathogenic bacteria such as *Porphyromonas gingivalis* [20]. Microbiota-induced periodontitis is one of the main factors that initiate oral malignancies, which are highly aggressive and characterized with aberrant MMP activity that drives tumor cell growth, angiogenesis, migration, invasion, and metastasis, but also on the contrary, certain MMP members function as tumor suppressors [48]. Beneath is an overview of the role of MMPs in driving microbiota dysbiosis-induced pathogenesis.

### 2.3. The Regulation of MMP Expression/Activity/Secretion by Microbiota–Host Interactions

Before delving into the contribution of local environmental microbiota to MMP-linked disease onset and pathogenesis, it is essential to recognize that nearly all infectious agents—including fungi, bacteria, and viruses—share a common virulence mechanism involving the induction of excessive MMP activity to facilitate their propagation into host tissues [49]. Secondly, it is essential to highlight the sites from which and mechanisms by which MMPs are expressed and secreted in response to altered microbial populations, as well as the microbial and immune factors that trigger local MMP activation in the colonized tissues. As explored across various independent microbiota environments, active MMPs primarily originate from three main sources: (1) First is the pathogens themselves, as they secrete MMP-like products (*e.g.*, thermolysin, pseudolysin, or karilysin) that function to destabilize tight junctions and degrade the ECM to promote infection propagation. These proteases also cleave and activate the locally present host MMPs to ensure sufficient ECM degradation [50]. (2) The active MMPs are also secreted by the colonized tissue in response to the infection. Specifically, the infected tissue stromal, epithelial, or mucosal cells upregulate MMP expression and secretion in the presence of secretory microbial antigens [*e.g.*, flagellin and phospholipase C (PLC)], metabolites, or toxins (*e.g.*, enterotoxin, TSST1, SSL5, and LPS) [51,52,53,54]. A detailed list is provided in Table 1. (3) Finally, there are immune response elements. Following pathogen recognition or sense of their toxins, innate immune cells (*e.g.*, PMNs or macrophages) secrete their MMP load to create gaps within the ECM that facilitate further immune cell infiltration [55,56]. The activation of the immune response also involves secretion of proinflammatory cytokines, such as IL-6, TNFα, and IL-1β, by the activated immune cells which, in turn, stimulate infected tissue cells to further express and secrete MMPs [57,58]. At this stage, oxidant moieties (*e.g.*, peroxynitrite) released by the immune cells react with ECM-embedded MMPs and trigger their activation [59,60]. Although the afore-mentioned mechanisms represent a common mode of MMP production and secretion in all infected tissues, the types of MMPs expressed and released differ across tissue types. Besides tissue specificity determinants that account for such differences, there are also MMP polymorphisms that define tissue MMP pools [61]. Polymorphisms in MMP promoters could broaden the transcription factor (TF) types that can bind and stimulate gene transcription or increase the affinity of a given TF to enter the binding site of the promoter [62].

To introduce further complexity, there are mechanistic regulatory pathways that involve TF-mediated control of MMP, as well as of TIMP, expression and activity, in response to microbiota dysbiosis. Of note, a basal MMP-9 expression level, sustained by SP-1 activity, may preserve symbiotic microbiota populations in colon epithelia; however, immune-induced MMP overexpression requires mainly nuclear factor kappa-light-chain-enhancer of activated B cells (NF-κΒ) and subsequent MAPK-activated AP-1 which can be activated in innate immune cells and tissue cells in response to shifts of microbial communities following stimulation by secreted cytokines, such as IL-1 or TNF-α [76,77]. Post-translational modifications of the TFs controlling MMP and TIMP gene expression can further affect the net outcome of gene transcription [78]. In addition to others, MMP genes contain binding sites in their promoters for AP-2, SP1, HIF, STAT, PDGF, and β-catenin. Cytokine (ILs, IFNs, TNF-α)*-* and growth factor (VEGF, PDGF, TGF-β, VEGF, HGF, EGF)-induced MMP expression is mediated by trans-activation of MMP promoters by recruitment of activated AP-1 [79]. It should be noted that in many cases, activation of a single TF is not adequate in controlling MMP expression, rather a TF interplay is required for an overall effect on MMP gene transcription [79].

Pathogenic bacteria have been shown to affect host epigenetics machinery and thus control host gene regulation [80]. While there is representative review over the topic, one mechanism involved in the process is enhanced acetylation of MMP promoters via the control of histone deacetylase (HDAC)/histone acetyltransferase balance in macrophages [81,82]. Also, most MMP and TIMP gene promoters contain CpG islands, making them prone to the action of DNA methyltransferases [83]. Besides the direct effects on MMP and TIMP genes, DNA methylation may also affect transcription of these genes by indirectly affecting NF-κΒ which controls their transcription [84]. Thus, although it is not able to retrieve direct evidence of microbiota dysbiosis in the epigenetics regulation of MMP expression in the diseases discussed in the following sections, the possibility that there actually are microbial-induced influences in epigenetic control of MMPs cannot be excluded. Even in this absence, microbiota dysbiosis and impaired immune function can lead to increased probability of infection with pathogenic strains that have already been demonstrated to influence epigenetic mechanisms, such as *Mycobacterium tuberculosis*.

Regulation of epigenetic mechanisms is not the sole mechanism for a pathogen to control host gene transcription. An alternative pathway is through the regulation of miRNA expression in host tissues or immune cells, a mechanism which pathogenic bacterial strains are using to control a host’s immune response on the merit of their survival [85]. While multiple miRNA-implicated pathways are employed in this pathogenic attempt, ranging from the regulation of Toll-like receptor (TLR)-mediated pathogen recognition to the regulation of pathogen clearance, the interplay between microbially affected host miRs and MMPs is of prime importance in microbial virulence and associated disease pathogenesis [86]. Describing all the evidence is not the scope of this review, which is thus limited on the topic by only presenting some examples and speculations, while proposing that a MMP regulatory loop is the key to underlying bacterial virulence.

Host miR machinery has been shown to be affected by pathogenic bacterial strains, such as *M. tuberculosis*, resulting in the modulation of immune responses in favor of the pathogens, via regulation of NF-κΒ activity, thus indirectly affecting MMP immune cell secretory load [85,87]. Further, MMP-9 has been shown to control miR-211 expression [88]. Interestingly, miR-211 is involved in LPS-induced endometritis, while serving as a limitation factor for NF-κΒ signaling, controlling the intensity of the local immune response [89]. Thus, it appears that there is an MMP-dependent transcription-mediated feedback loop mechanism by which bacteria initially utilize host MMP sources to accomplish barrier breakdown and propagation into distant colonizing areas, while later avoiding the aberrant immune response that will compromise their survival [72]. During the host MMP secretory boost, which can also be triggered selectively by pathogenic bacteria forcing RECK downregulation to host tissues, the pathogens can protect themselves against proteolysis by utilizing host-derived MMP inhibitors, such as thrombospondin or A2M [90,91]. Recently, direct support of host MMP exploitation came from a study where *Staphylococcus aureus*-induced miR-939 in patients’ keratinocytes was correlated to increased invasiveness in a mouse model of atopic dermatitis [92]. Nearly all documented bacteria-induced immunomodulatory miRs can target MMP mRNA sequences and affect host MMP expression, and some examples are miR-29b, miR-146, and miR-155 [93,94,95]. Targeting of this feedback loop may explain, at least in part, the action of probiotics in restoring beneficial strain growth and protecting against disease. For example, with regards to gut microbiota, the probiotics *Lactobacillus paracasei* and *Lactobacillus casei* limit the infection of *Listeria monocytogens* by restoring the pathogenically compromised levels of miR-192, miR-200b, and miR-215, all of which have been shown in independent studies to target MMPs [96,97]. Similarly, the only MMP inhibitor that has been approved by the Food and Drug Administration (FDA) and reached clinical practice, the antibiotic doxycycline, exerts suppressive effects over MMP expression that are partly mediated (in addition to zinc-chelating properties) by inductions of miRs that target the MMP transcripts [98].

Finally, it is important to note that probiotic-induced restoration of immunoregulatory miRs that function via MMP targeting may expand the use of probiotics as adjuvant treatments for diseases irrelevant to microbe-induced pathogenesis, such as asthma or cancer [99,100]. In a similar fashion, tetracyclines (*e.g.*, doxycycline) that exhibit potent MMP inhibitor properties have proved promising for the treatment of diseases pathologically correlated to aberrant MMP activity, as evidenced by in vivo studies as well as by studies of patients [101,102].

## 3. The Dynamic Relationship Between MMPs and the Oral Microbiome: The Implications for Oral Health and Disease

The oral microbiome (OM) is defined as the collective genetic material derived from the oral colonizing microbiota. Oral microbiota consists of microorganisms of all taxa and is present in the form of a biofilm covering all oral surfaces, including the hard and soft denticular tissues (teeth and gingiva) and the oral mucosa (tongue, cheeks, tonsils, and hard and soft palate) [6,103]. The natural role of the OM is to promote and sustain oral homeostasis, which is facilitated through natural competition with pathogens for nutrient utilization. It is estimated that over 700 bacterial species are present in the OM with some of them known to protect against acidification and pathogens through the secretion of microbicidal substances, while others serve as support (scaffolds) for the growth of anaerobes [103,104,105]. The interaction of oral bacteria with the oral epithelium shapes the structural and functional changes in the oral cavity and drive innate immunity by triggering epithelial and mucosa cells to produce and secrete mucus and antimicrobial compounds [106]. Perturbations of the populations that comprise the OM result in dysbiosis which is clinically manifested in the form of oral disease, such as periodontitis, caries, and candidiasis. Further, OM imbalances are correlated with the onset of systemic diseases, such as hypertension and cancer [104,107].

Fungi normally present in the OM serving as scaffolds for bacterial growth may progress as opportunistic infectants once the host’s immune defense is compromised [108]. OM composition and function are variable and dependent on various factors, including diet, the presence of irritants, medications (mainly antibiotics and immunosuppressants), diseases and poor hygiene, the latter leading to dental plaque formation, a bacterial biofilm that affects OM populations [109,110]. The correlation between OM dysbiosis and systemic disease is reciprocal; systemic disease, *e.g.*, diabetes, can affect oral pH and determine OM composition [110]. Further, a less efficient oral–gut barrier, including a less acidic gastric fluid, often seen in neonates and the elderly, allows microbial translocation from the oral cavity to the intestines and can act as the trigger of diseases such as colon cancer [111,112]. Thus, translocation of acid-resistant bacterial strains from mouth to gut may lead to intestinal inflammation and IL-9-triggerred systemic disease [113].

MMPs are major immune regulatory participants in the oral cavity, acting either as proinflammatory or anti-inflammatory mediators. In gingiva, MMPs are endogenous components of the dentin fibrillar network, possessing functional roles in physiological processes of enamel maturation, dentin mineralization, alveolar bone regeneration, tooth maturation and movement, and inhibition of dentinogenesis [114,115]. Inflammation-related MMP secretion is often accompanied by the secretion of IL-8, IL-1β, TNF-a, osteoprotegerin, PGE2, and the receptor activator of NF-kB ligand (RANKL) by both immune and non-immune cells during the initiation of the innate immune responses [116]. Secreted MMP-8 and MMP-9 bind on TIMP-1 on the surface of infiltrated PMNs, where they become anchored and promote PMN-induced proteolysis of the ECM so the migration of PMNs is assisted [117]. On the contrary, MMP-8 can alleviate inflammatory responses to injury by cleaving chemokines, a mechanism possibly involved during immune evasion of oral malignancies [118].

### 3.1. MMPs in Oral Microbiome Dysbiosis: Implications for Periodontal Disease and Dental Caries

Periodontitis is a disease affecting the deeper layers of gingiva (gums), teeth and the surrounding (alveolar) bone, often manifesting following severe gingivitis. The cause of periodontitis is the aberrant chronic inflammatory response due to irritants, such as smoke, caries, or pathogenic microbes. The OM plays a pivotal role in disease progression, as periodontitis is the result of dysbiosis of the OM where beneficial strains are depleted and replaced by anaerobic strains that constitute the plaque (*e.g.*, *T. denticola*, *T. forsythia*, and *P. gingivalis*), including methanogenic archaea and bacteriophages [119,120]. The most detrimental consequences of periodontitis are presented at age extremes; advanced stages of periodontitis in the elderly may be associated with mouth malignancies, whereas in children, it can lead to the development of Noma disease (cancrum oris), a gangrenous stomatitis involving necrotic lesions in gums, teeth, and facial skin which can lead to 90% mortality rates [121,122].

At a molecular level, the key mediator of periodontitis is IL-17 and RANKL. IL-17, specifically, is secreted by both innate (macrophages, dendritic cells, NK cells, monocytes, neutrophils, etc.) and adaptive (B and T cells) immune cells [123,124]. At disease onset, monocytes encountering *P. gingivalis* and *A. actinomycetemcomitans* secrete IL-17 which promotes the secretion of antimicrobial compounds, as well as the recruitment of NK cells, type 3 innate lymphoid cells, and neutrophils [124,125]. RANKL expression is elevated in the recruited cells and accounts for the drive of differentiation of osteoclast precursors to mature osteoclasts that will facilitate bone resorption, while MMPs will catalyze tissue destruction [126].

The oral inflammatory response is regulated by the levels of MMPs and TIMPs [127]. In the oral cavity, MMPs are secreted by many cell types, including keratinocytes, fibroblasts, macrophages, and neutrophils [128]. Overexpression and overactivation of MMPs result in periodontal tissue destruction, and profound MMP-8 and MMP-9 elevations are associated with periodontitis severity [129]. Furthermore, MMP gene polymorphisms constitute a predisposing parameter for the development of periodontitis and salivary MMPs provide a prognostic biomarker tool [130]. 

OM dysbiosis-mediated periodontitis is characterized by an increased expression of MMPs. In addition to the MMPs produced and secreted by innate immune cells following pathogen encounter, MMPs are synthesized and secreted by bacteria themselves, as well as by the colonized tissue [131]. As such, production of bacteria-secreted proteases, as well as PMN secretion of myeloperoxidase following the phagocytosis of the bacteria, induce MMP production [132]. Gram-negative bacteria of the *Prevotella intermedia* species have been shown to upregulate MMP-1, MMP-8, and MMP-9 in human periodontal cells [133,134]. On the contrary, there are periodontitis-negative bacterial strains within the OM, such as *Corynebacterium matruchotti*, that counteract MMP-9 elevation in oral host tissues [135].

Carious lesions are attributed mainly to bacterial acid-induced demineralization of enamel and dentine, as well as to the abnormally high levels of MMP-8 in the saliva. MMP-2, MMP-8, MMP-9, and MMP-20 have been implicated in caries-induced dentine matrix destruction, as well as in periodontitis [136,137]. Recently, gene polymorphisms of lactoferrin and MMP-13 were identified as predisposing risk factors for caries development and caries-preventive interventions have been developed based on MMP inhibition [138,139].

Acids from mainly U32 protease-positive bacteria, such as *Streptococcus mutans*, *Bifidobacterium*, *Lactobacillus*, *Dialister* spp., and *Filifactor* spp., demineralize dentin and expose the tooth roots to a protease-rich environment, but also directly activate the host’s dentin-embedded and salivary matrix MMPs (*e.g.*, MMP-2) and cathepsins [140,141,142]. These bacteria depend on carbohydrate metabolism and therefore, dietary intake of sugars greatly affects caries progression and cavitation. Acidogenic bacterial virulence is mediated by the synthesis and secretion of glucosyltransferases (GTFs), enzymes that function to make the plaque matrix adhesive and insoluble, so it can then allow colonization of different bacterial strains. GTFs also bind the surface of other microorganisms and convert them to glucan producers, providing a pathological symbiosis pattern. Interestingly, glycosylation of the extracellular matrix metalloproteinase inducer, EMMPRIN, which is the upstream activator of MMP-2 and MMP-9, increases its efficiency in activating its substrates and thus, targeting GTFs is a promising approach towards caries prevention [143,144].

### 3.2. Microbiome-Mediated Modulation of MMP Activity in Oral Squamous Cell Carcinoma

Oral squamous cell carcinoma (OSCC) is the most common type of head and neck malignancy and the deadliest form of oral cancer, as it is frequently metastasizing to adjacent tissues and local lymph nodes, as well as to peripheral organs (liver, lungs, and bones) [145]. OSCC is initiated in the oral mucosa; a precancerous oral mucosa demonstrates specific clinical characteristics, the examination of which provides a valuable tool for OSCC prediction [146]. In addition to genetic predisposing determinants of OSCC evolution, which include growth factors (mainly EGF and TGF-β) and corresponding receptors, OSCC can be environmentally acquired by the exposure of oral epithelia to carcinogenic chemicals (*e.g.*, tobacco smoke) and heavy metals, where multiple signaling cascades are involved, as well as by infectious agents, such as HPV [147,148,149]. With regard to the latter, an association has been an established between OM dysbiosis and the incidence of OSCC; approx. 7–15% of OSCC cases are attributed to OM dysbiosis [150,151]. A detailed list of pathogens implicated in OSCC is given in the recent review of Sukmana and colleagues [152].

The bacterial strains recognized to contribute to OSCC evolution are *Actinomyces*, *Clostridium*, *Cpnocytophaga gingivalis*, *Prevotella* spp., *Fusobacterium* spp., *Streptococcus* spp., *Peptostreptococcus* spp., and *P. gingivalis*. *F. nucleatum* and *P. gingivalis* have gained special attention as they are particularly involved in OSCC progression [153,154]. It should be noted that the presence of an individual species is not adequate for OSCC risk conference; rather, this demands complex interactions of multiple species with the oral mucosal surfaces [155]. For example, the tumorigenic effects of *F. nucleatum* are elicited partly through their coherence with other bacterial strains as well as with *Candida albicans* [156]. The OM-mediated causative factors that drive OSCC carcinogenesis include the synthesis and secretion of genotoxic peptides and chronic and persistent inflammation. Lifestyle factors, such as the combination of smoke with alcohol use and poor hygiene, promote OM dysbiosis by causing persistent inflammation [157]. Restoration of optimal OM balance using probiotics is a promising preventive strategy against OSCC development, as these can re-establish beneficial bacterial species populations and ameliorate the oncogenic proinflammatory response occurring in the oral tissues [135]. Further, probiotics may be used as adjuvant therapy to increase the efficiency of current chemotherapeutic protocols [158].

Various mechanisms are involved in altered microbiota: induced OSCC oncogenesis, including the eliciting of direct mutagenic effects induced by microbes or indirect effects mediated by the aberrant inflammatory response (involving oxidative and nitrosative stress-induced mutagenesis, as well as cytokine-mediated) on the epithelial mucosa [159]. Further, the altered bacterial strains can modulate epithelial mucosal cell proliferation and apoptosis by triggering the activation of NF-kB and influencing the epigenome, triggering oncogenic gene transcription [160,161]. Byproducts of bacterial metabolism, such as lactic acid, acetaldehyde, and pyruvate/indole, can promote or sustain OSCC oncogenesis [162]. *F. nucleatum* can directly trigger epithelial-to-mesenchymal transition (EMT) activation, while *P. gingivalis* can assist OSCC cells to evade immunity by directly promoting T-reg differentiation and thus, inhibiting effector T-cell expansion, as well as metastasis, through the destruction of the basement membrane barriers [163,164]. Similarly, *C. gingivalis* has been shown to secrete hydrolytic enzymes that promote tissue and bone digestion, as well as aminopeptidase, a collagen fiber-degrading enzyme, thus promoting metastasis potential for OSCC cells [153]. Finally, acidification of the tumor microenvironment by tumor-colonizing bacteria-secreted acids, such as lactic acid from *Lactobacilli* and *P. stomatis*, may sustain tumor growth and potentiate metastasis [165,166].

Since OSCC demonstrates such high metastasis rates, it is reasonable that it is highly dependent on MMP function to degrade the ECM and invade nearby and distant tissues. Indeed, OSCC is associated with increased expression of MMPs and gene polymorphisms in MMP genes are associated with different tumor properties, as well as with differential response to therapy [167,168]. A recent bioinformatics analysis revealed that MMP-1 can be used as a head and neck cancer biomarker, while a recent Mendelian randomization analysis indicated that MMP-9 constitutes a risk factor for OSCC development [169,170]. Similarly, saliva MMP-13 has also been suggested as an OSCC prognostic biomarker. MMP-11 drives OSCC migration and metastasis via activation of the FAK/Src pathway and is associated with poor prognosis [171]. Similarly, ERK-mediated upregulation of MMP-2 promotes metastasis in OSCC patients [172].

The inflammatory response per se is a causative indirect factor for MMP level elevation, as IL-8 and TGF-β1 secreted by immune cells have been shown to activate MMPs in OSCC cell lines [173]. However, the OM dysbiosis environment positively affects MMP regulation in favor of cancer cells. Dentilisin, a protease secreted by *Treponema denticola*, has been shown to activate MMP-8 and MMP-9, potentiating ECM remodeling and metastasis [68,174]. For instance, MMP-2 and MMP-9 are conceived as major regulators of OSCC progression, promoting tight junction destabilization, and their levels are increased in the saliva of OSCC patients compared to healthy individuals [175,176].

**Figure 2:**
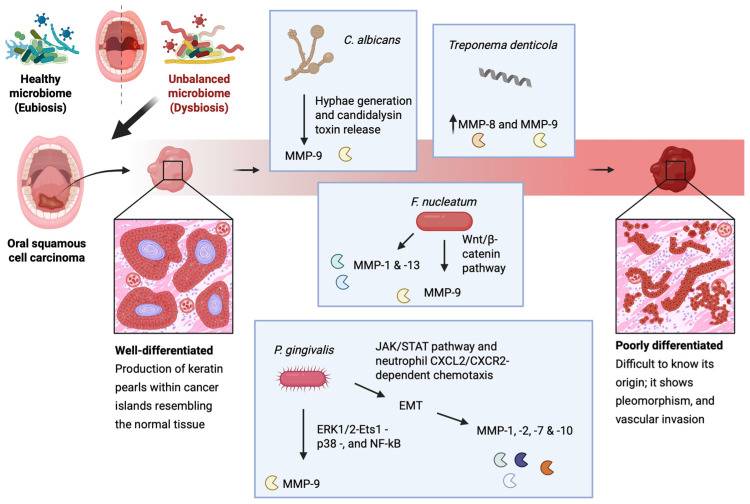
A schematic representation of the microbiota-induced expression of MMPs during OSCC. In oral dysbiosis, *P. gingivalis* exposure stimulates oral cancer cells to upregulate MMP-1 and MMP-2, enhancing their invasiveness. It promotes MMP-9 expression through ERK1/2-Ets1-p38 and NF-κB signaling, while also driving EMT via the JAK/STAT pathway and neutrophil CXCL2/CXCR2-dependent chemotaxis. Additionally, it induces the expression of EMT-related MMP-1, MMP-2, MMP-7, and MMP-10. *F. nucleatum* activates MMP-9 via Wnt/β-catenin signaling, destabilizing adherence junctions, and increases MMP-1 and MMP-13 in noncancerous epithelial cells. Finally, *C. albicans* promotes MMP-9 activation through hyphae formation and candidalysin release. This figure was created with BioRender.com.

Exposure to *P. gingivalis* can stimulate oral cancer cells to upregulate MMP-1 and MMP-2 which are linked to their enhanced invasive properties [177]. This bacterial strain is considered as a major OM-related EMT inducer in OSCC, as it has been demonstrated that it can promote ERK1/2-Ets1-p38- and NF-kB-mediated MMP-9 expression, as well as EMT induction via the JAK/STAT pathway and neutrophil CXCL2/CXCR2-dependent chemotaxis [178,179]. In addition, it can stimulate EMT induction-relevant MMP-1, MMP-2, MMP-7, and MMP-10 expression [177,180,181]. Similarly, *F. nucleatum* activates MMP-9, although it does so via activation of the Wnt/β-catenin pathway following liberation of β-catenin from the cell’s surface adherens junctions and their subsequent destabilization [182,183]. In host noncancerous epithelial cells, *F. nucleatum* has been shown to upregulate the expression of MMP-1 and MMP-13 as well [184]. Lastly, *C. albicans* also potentiates MMP-9 activation following hyphae generation and candidalysin toxin release [70,71]. As seen, MMP-9 plays a pivotal role in OSCC progression and is often associated with poor prognosis, and can therefore be used as an OSCC prognostic biomarker [176,185] (Figure 2).

## 4. The Role of MMPs in Gut Microbiome Dynamics

The gastrointestinal tract hosts the largest population of microorganisms in the human body [186] and these exert various functions to ensure systemic homeostasis, ranging from nutrient absorption to pathogen antagonism and the shaping of the immune system [187,188]. The intestinal wall is a highly complex structure that plays a critical role in maintaining the integrity and functionality of the intestinal epithelium [189]. It serves a dual function: acting as a protective barrier against pathogen infiltration and colonization while regulating intestinal permeability—the controlled passage of water, nutrients, and ions across the epithelial layer [190]. This permeability is tightly regulated through coordinated interactions between epithelial cells, crypt-associated signaling pathways influenced by mesenchymal cells, and extensive communication with components of the ECM [34]. Under normal conditions, host–microbiota interactions provide gut immune tolerance [188]. As such, minor epithelial damage triggers a cascade of repair mechanisms. Within this process, the tissue mesenchyme plays a key role in regulating ECM proteolysis through enzymes such as MMPs, which facilitate tissue remodeling and epithelial restoration [191,192]. The immunomodulatory effects of intestinal microflora may also account for immune cell-derived MMPs [188]. However, in pathological conditions, significant alterations in the gut microbiome composition led to the release of microbial signals that compromise the integrity of the gut barrier and activate immunoregulatory mechanisms. Inflammatory conditions affecting the intestine disrupt the delicate balance of epithelial–ECM interactions, resulting in excessive proteolytic activity and increased intestinal permeability, highlighting the importance of MatrixBiome [193].

### 4.1. The Role of MMPs in Gut Microbiome Dynamics During Inflammatory Bowel Disease

Inflammatory bowel disease (IBD) is a chronic, non-infectious, and relapsing–remitting inflammatory disorder of the gastrointestinal tract, primarily comprising Crohn’s disease (CD) and Ulcerative Colitis (UC). Although the exact causes remain uncertain, a combination of genetic susceptibility, environmental factors, microbial dysbiosis, and immune system dysfunction are known to drive disease onset and progression [189]. Both CD and UC present with overlapping symptoms such as abdominal pain, fever, diarrhea, vomiting, rectal bleeding, weight loss, and anemia [194]. However, they differ in their pathological patterns and treatment strategies. CD is distinguished by discontinuous, transmural inflammation that can affect any part of the gastrointestinal tract, whereas UC is characterized by continuous inflammation limited to the mucosal and submucosal layers of the colon [195]. Dysbiosis has also been identified in fecal samples in IBD, which is characterized by the reduced abundance of the phylum Firmicutes (*e.g.*, *Faecalibacterium*, *Roseburia*, and *Ruminococcus*) and an increase in the phylum Proteobacteria (*e.g.*, *Enterobacteriaceae*) [196,197]. These changes are more prominent in CD than UC [198].

When the epithelial–interstitial barrier is compromised due to injury, an inflammatory response is triggered, leading to the recruitment of immune cells to the wound site. ECM components play a crucial role in modulating immune responses and facilitating tissue repair. Initially, epithelial cells release cytokines that attract neutrophils to the damaged area. However, prolonged cytokine release due to unresolved epithelial injury results in neutrophil degranulation and the secretion of ECM-degrading enzymes [199]. Among these, MMPs are key regulators of tissue remodeling. They degrade ECM components, promote cell migration, and release ECM-derived proteins and glycoprotein fragments, which further recruit immune cells and drive tissue turnover [200]. Additionally, MMP expression is upregulated in stromal cells, particularly myofibroblasts. In IBD, the equilibrium between ECM degradation and deposition is disrupted. Beyond their proteolytic activity, MMPs contribute to excessive ECM accumulation, characterized by increased expression of collagens (types I, III, and V) and fibril-associated collagens with interrupted triple helices (FACITs) such as types XII, XVI, and XIX [192]. The excessive deposition of ECM due to the failing of wound healing leads to sustained inflammation and fibrosis [201]. The increased activity of MMPs in in vitro and in vivo IBD models and IBD patients has been adequately demonstrated and addressed [202,203],; however, the influence of microbiota on MMP activity has not been elucidated. The microbiome can affect the MMP activity directly, by the secretion of bacterial-derived proteases, or indirectly, via the secretion of several bioactive metabolites that induce the expression of MMPs from epithelial and subepithelial host tissues. Here, we mainly report the impact of indirect communication between microbiome and host cells via metabolite secretion.

The gut microbiome produces various metabolites, such SCFAs, secondary bile acids, tryptophan metabolites, and polyamines, which can modulate MMP expression and influence intestinal inflammation and tissue remodeling. Several of these metabolites can also be delivered by food sources or be synthesized endogenously; however, microbiota-mediated production may facilitate localization/retention of these factors to specific tissues (*e.g.*, mouth or CNS following gut escape), augment their local concentrations and disturb other microbiota or exhibit altered potency due to the co-presence of additional metabolites.

SCFAs are metabolic byproducts generated by gut microbiota through the fermentation of dietary fiber [204]. The primary SCFAs—acetate, butyrate, and propionate—can collectively reach concentrations exceeding 100 mM in the intestines. Acetate and propionate are predominantly produced by Bacteroidetes, while Firmicutes are the main contributors to butyrate production [205]. Studies have proved that metabolites modify the expression levels of MMPs in host tissue in IBD. For example, propionate and butyrate significantly attenuated IL-1 beta- and TNF-α-induced MMP-1 and MMP-3 secretion in human colon myofibroblasts [63], while lactate has been shown to suppress TNF-α-induced MMP-9 expression in IBD [65]. The suppression of MMP-9 protects tight junction proteins from disruption by inhibiting NF-κB activation via GPR81 in vitro and preserves intestinal epithelial barrier integrity against dextran sodium sulfate (DSS)-induced in vivo models [65]. Butyrate decreases proinflammatory cytokine expression via inhibition of NF-κB activation and IκBα degradation, thereby reducing ECM degradation and intestinal barrier disruption in IBD [206]. In addition, prolonged and low-dose butyrate concentration in gut increases pro-MMP-2 and pro-MMP-9, but the parallel increase in TIMP-1, TIMP-2 expression prevents the formation of the active MMP-2 form [66]. Interestingly, in a model of bacteria-induced colitis, the microbial community of MMP-9−/− mice contributes to reduced levels of *C. rodentium*, preventing a reduction in the microbial diversity associated with infection [77].

### 4.2. Role of MMPs in Gut Microbiome in Colorectal Cancer

Besides SCFAs, secondary bile acids (SBAs), such as deoxycholic acid (DCA) and lithocholic acid (LCA), are produced by gut microbiota through the metabolism of primary bile acids (BAs) in the intestine. These SBAs interact with nuclear and membrane receptors TGR5, FXR, and PXR, influencing inflammation, gut barrier function, and MMP expression [207,208]. BAs are essential for digestion, lipid absorption, and maintaining intestinal epithelial homeostasis. In the liver, primary BAs are conjugated with glycine or taurine and stored in the gallbladder. Upon secretion into the intestine, they aid in fat digestion. High-fat diets increase BA secretion, leading to higher colonic primary BA concentrations. Most conjugated BAs are reabsorbed in the ileum via the apical sodium-dependent bile salt transporter, but 5–10% escape reabsorption, becoming substrates for microbial metabolism. Gut bacteria, including *Clostridium*, *Enterococcus*, *Bifidobacterium*, *Bacteroides*, and *Lactobacillus*, possess bile salt hydrolase (BSH), which deconjugates BAs [209]. This microbial activity leads to the formation of secondary BAs, which have been linked to colon carcinogenesis [210,211].

Interestingly, DCA has been shown to induce CXCL8 expression in colorectal cancer (CRC). CXCL8 expression correlated with β-catenin localization in epithelial cells of adenomas, as well as in endothelial cells and neutrophils. DCA-mediated CXCL8 induction occurs in initiated colonic epithelium and neutralizing CXCL8 could reduce the invasive potential of tumors [212]. DCA promotes invasion in esophageal squamous cell carcinoma through the upregulation of MMP-10 [74], whereas in CRC, DCA promotes vasculogenic mimicry formation and EMT through VEGFR2 activation [213]. The other SBA, LCA, has been reported to induce urokinase receptor (uPAR) expression via Erk-1/2 and AP-1 pathways, which stimulate the invasiveness of human colon cancer cells [214].

**Figure 3:**
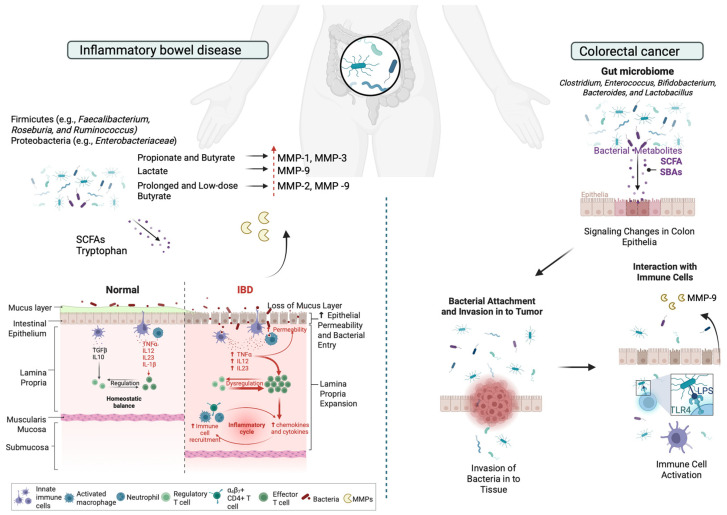
Schematic representation of the microbiota-induced expression of MMPs in ECM remodeling during IBD and CRC. In IBD, Bacteroidetes mainly produce acetate and propionate, while Firmicutes generate butyrate. SCFAs regulate MMP expression in IBD; propionate and butyrate reduce IL-1β- and TNF-α-induced MMP-1 and MMP-3 secretion, while lactate suppresses TNF-α-induced MMP-9 via GPR81, preserving intestinal barrier integrity. Butyrate inhibits NF-κB activation, reducing ECM degradation, though prolonged low-dose exposure increases pro-MMP-2 and pro-MMP-9 alongside TIMP-1 and TIMP-2, preventing active MMP-2 formation. In CRC, LPS, a key component of Gram-negative bacteria like *S. marcescens* and *E. coli*, contributes to IBD by activating the TLR-4/NF-κB pathway, leading to gut barrier dysfunction and promoting CRC. Elevated MMP-9 levels disrupt tight junctions, increasing intestinal permeability. This figure was created with BioRender.com.

Lipopolysaccharide (LPS), a component of the outer membrane of Gram-negative bacteria, such as *S. marcescens* and *E. coli*, plays a significant role in the pathogenesis of IBD. Chronic inflammation leads to increased levels of LPS, which activates the TLR-4/NF-κB signaling pathway, leading to gut barrier dysfunction and encouraging CRC development [215]. Elevated MMP-9 levels have been associated with the disruption of tight junctions in intestinal epithelial cells, leading to increased intestinal permeability and barrier dysfunction [216]. LPS-induced MMP-9 expression and cell migration is promoted via inhibition of the ROS/ERK pathway in colorectal cancer [217]. This compromised barrier allows for the translocation of bacteria and antigens, further exacerbating inflammation (Figure 3).

## 5. Unraveling the Role of MMPs in the Gut–Body Connection: Interactions Between the Gut, Brain, and Skin

### 5.1. Exploring the MMP–Gut–Skin Axis: The Implications for Health and Disease

#### 5.1.1. Exploring the Role of MMPs in Healthy Skin Tissue

The skin, as the largest organ of the human body, is home to a diverse and dynamic ecosystem composed of bacteria, fungi, viruses, and archaea that play a crucial role in maintaining skin homeostasis, immune regulation, ECM remodeling, and defense against pathogenic colonization [218,219,220]. Skin’s foremost function is to provide a physical, immune, and chemical barrier between the body and the outside environment, including pathogens [220,221,222]. However, any imbalance in the composition of the skin microbiome can potentially influence MMP activity and contribute to skin disorders such as psoriasis, acne and melanoma [219,220]. The MatrixBiome’s gut–skin axis highlights how gut dysbiosis may also contribute to skin conditions [223].

Dysbiosis in the gastrointestinal tract is linked to inflammatory diseases, including skin conditions such as psoriasis, acne vulgaris, and skin cancer [223]. The gut microbiome functions as the body’s largest endocrine organ, producing over 30 hormone-like compounds, including SCFAs, SBAs, cortisol, and neurotransmitters such as gamma-aminobutyric acid, serotonin, dopamine, and tryptophan [224]. These bioactive compounds enter the bloodstream and influence distant organs, including the skin [224]. This highlights a bidirectional relationship between the individual components of gut and skin axis, primarily driven by immune system modulation in response to gut dysbiosis [225].

In healthy skin, MMPs regulate essential processes, including normal skin turnover, wound healing, immune surveillance, and antimicrobial defense [12]. More importantly, it is known that MMPs, such as MMP-1 and MMP-9, degrade collagen and elastin, allowing controlled ECM turnover [226]. Moreover, it is known that in normal healthy conditions, commensal bacteria help to modulate MMP activity, ensuring balanced ECM turnover and skin homeostasis [227,228]. Overexpression of MMPs, especially MMP-9, has been observed in various pathological skin conditions [12,226], while dysbiosis can contribute to impaired skin barrier repair and increased susceptibility to inflammation, potentially also leading to altered MMP expression [227,229]. By maintaining a stable presence in the skin microbiome, commensal bacteria contribute not only to immune surveillance but also to the preservation of skin barrier function, reducing the risk of pathogenic colonization and inflammatory skin diseases [230]. For example, certain commensal bacteria, like *Staphylococcus epidermis*, have been shown to promote the expression of antimicrobial peptides, while *Corynebacterium* species contribute to lipid metabolism, influencing ECM stability and MMP regulation [231,232]. The presence of *Cutibacterium acnes* at physiological levels regulates MMP expression by influencing keratinocyte differentiation and inflammatory pathways, through the secretion of SCFAs, such as butyrate [220,233].

#### 5.1.2. The Implications of MMPs in the Gut–Skin Axis in Inflammatory Skin Diseases

Skin inflammatory diseases, such as psoriasis and acne vulgaris, are characterized by chronic inflammation driven by an abnormal immune response, leading to tissue damage and disruption of normal physiological processes [234]. In detail, psoriasis is a chronic immune-mediated inflammatory disorder with autoimmune characteristics, affecting both the skin and joints through complex immune dysregulation and aberrant keratinocyte proliferation [235,236]. Psoriasis presents as well-demarcated erythematous plaques with silver-white scales, predominantly on the knees, elbows, scalp, umbilicus, and lumbar region [235]. The pathogenesis of psoriasis, which is a chronic Th17-driven inflammatory skin disease, involves dysregulated crosstalk between the innate and adaptive immune systems, mediated by the IL-23/IL-17 axis, TNF-α and NF-κB signaling, leading to excessive keratinocyte proliferation and systemic inflammation [237,238]. Inflammation is accompanied by increased MMP expression, with monocytes, macrophages, and neutrophils secreting various MMPs to facilitate immune cell migration [239]. MMP-19 plays a key role in T-cell-mediated immunity, as its absence impairs inflammatory cell influx, cytokine production, and keratinocyte proliferation [239]. Psoriasis is also linked to increased colonization by *C. simulans* and *C. kroppenstedtii*, alongside a reduction in *Lactobacillus*, *P. acnes*, and *Corynebacterium* spp., which may partake in regulatory functions [240].

The pathogenesis of psoriasis also involves key physiological processes regulated by MMPs, such as degradation of ECM and the basement membrane components [241]. MMPs facilitate tissue remodeling, cell migration, angiogenesis, and epithelial apoptosis, all of which relate to psoriasis progression [241]. MMPs regulate keratinocyte migration by degrading adhesion molecules and remodeling the ECM. MMP-2 and MMP-9 inhibition reduces keratinocyte mobility, while MMP-19 promotes migration by cleaving laminin-5 γ2 [241,242]. It is important to mention that in psoriatic patients, serum concentrations of MMP-2, MMP-3, and MMP-9 are significantly higher, without an accompanied overexpression of TIMP-1 or TIMP-2 [243]. However, excessive MMP-9 disrupts ECM integrity, impairing migration and delaying wound healing [241,244]. MMPs also regulate epidermal adhesion by cleaving key structural proteins. MMP-9 disrupts hemidesmosomes by degrading the β4 integrin ectodomain, compromising epidermal integrity and contributing to pustular psoriasis [245,246]. Additionally, MMP-9 is highly expressed in psoriatic epidermis, further weakening adhesion structures and exacerbating disease pathology [241]. Additionally, angiogenesis in psoriatic skin is driven by elevated VEGF, HIF1α, MMP-2, and MMP-9 levels, which correlate with increased capillary formation [247]. VEGF stimulation upregulates MMP-2 and MMP-9, promoting endothelial cell organization into capillary-like structures, while MMP inhibition suppresses this process [248]. MMP-12 further contributes to angiogenesis by shedding the uPAR, facilitating endothelial cell invasion and proliferation, whereas its inhibition impairs capillary morphogenesis [249,250].

Beyond the skin microbiome, the gut–skin microbiome axis is an emerging factor for the progression of psoriasis, since intestinal dysbiosis may trigger the progression of the disease. Common findings include reduced *Bacteroides* and *Akkermansia* spp., alongside increased *Bacillota* and *Actinomycetota phyla* [251,252,253]. This imbalance promotes bacterial translocation and systemic inflammation, mirroring mechanisms observed in inflammatory bowel disease and Crohn’s disease [238,254]. It is known that the lowered or minimal SCFA production from the gut microbiota of psoriatic patients is similar to patients with intestinal diseases [255,256]. Gut-derived SCFAs exert their effects by activating G protein-coupled receptors (GPCRs) expressed on various skin cells, such as keratinocytes, as well as immune cells like leukocytes and neutrophils, thereby modulating tissue metabolism and function [257,258,259]. SCFAs play a regulatory role in psoriasis by modulating T regulatory activity and promoting keratinocyte differentiation, with lower SCFA levels correlating with disease severity [259,260,261].

Acne vulgaris is a chronic inflammatory disorder of the pilosebaceous unit (hair follicle, hair shaft, and sebaceous gland), primarily arising during puberty but also affecting adults [262]. Its pathogenesis involves alteration in sebum quantity and its free fatty acid composition (dysseborrhea), dysregulation of hormones, abnormal follicular keratinization, and *Cutibacterium acnes* (formerly known as *Propionibacterium acnes*) proliferation, which collectively alter the cutaneous microenvironment and trigger inflammatory responses that drive lesion progression [263,264]. Th17 lymphocytes play a pivotal role in acne pathogenesis by triggering inflammatory cascades that activate cytokines, leading to neutrophilic infiltration and pilosebaceous follicle inflammation. These lymphocytes stimulate various cells to produce proinflammatory mediators, including IL-6, TNFα, IL-1β, PGE2, nitric oxide, and MMPs [265]. Moreover, there are indications that *C. acnes* enhances the activity of various MMPs, including MMP-1, MMP-9, and MMP-13, which have been detected in the sebum of acne patients [266]. Significantly elevated MMP-9 levels were also found in the blood of acne patients and a correlation between MMP-9 levels and the extent of facial inflammatory lesions was revealed, though no direct link was established between MMP-9 and scarring [267]. The NF-κΒ pathway is highly active in acne lesions, with *C. acnes* contributing to inflammation by inducing TLR-2 and TLR-4 expression [268]. Acne is associated with an imbalance in the skin microbiota than a mere overgrowth of *C. acnes* [264]. While *C. acnes* is a commensal omnipresent in the sebaceous follicles and at lower quantities at the skin surface, acne is associated with specific phylotypes, particularly IA1, which thrive in hyperseborrheic environment [264,269]. The loss of diversity triggers innate immune activation and inflammation, as IA1 monocultures stimulate immune responses [270]. Additionally, *C. acnes* strains differentially modulate CD4+ T-cell responses, promoting Th17 activation and interferon-gamma production, further contributing to acne-related inflammation [222,271]. Other microbes may contribute to acne pathogenesis, such as *C. granulosum*, which is found in acne lesions and exhibits greater virulence than *C. acnes* [271]. *Malassezia*, with higher lipase activity, hydrolyzes sebum triglycerides into free fatty acids, promoting comedone formation and hyperkeratinization [271]. It also stimulates cytokine release from monocytes and keratinocytes, and antifungal treatments have improved resistant acne cases [271,272]. *S. epidermis*, more abundant in acne patients, regulates *C. acnes* growth and reduces inflammation via staphylococcal lipoteichoic acid, suggesting a potential role in acne recovery [273,274].

Recent studies highlight the gut microbiota’s role in skin homeostasis, influencing systemic immunity and directly affecting the skin through microbial translocation and metabolite production [275,276]. SCFAs, such as propionic acid, modulate skin microbiota and cutaneous immunity [271,277]. Emerging research links gut dysbiosis to acne, particularly through the mTOR pathway, which regulates cell growth, fat metabolism, and inflammation [278]. Studies have shown that acne patients exhibit reduced gut microbiota diversity, with lower levels of *Lactobacillus*, *Bifidobacterium*, and *Butyricicoccus*, which are essential for gut barrier integrity and immune regulation [279]. *C. acnes* activates TLR-2, triggering the release of proinflammatory cytokines such as IL-1, IL-6, IL-8, and TNF-α, leading to skin inflammation. As previously mentioned, IL-6 acting through STAT3 can upregulate MMP-9 levels, leading to a compromised skin barrier. SCFAs may mitigate this response by inhibiting histone deacetylases—HDACs [280]. Additionally, butyrate enhances the expression of filaggrin and transglutaminase-1, promoting the terminal differentiation of normal human epidermal keratinocytes and maintaining skin homeostasis through HDAC inhibition [281].

#### 5.1.3. Implications of MMPs in Gut–Skin Axis in Skin Cancer

Cutaneous malignancies are principally classified into melanomas and non-melanoma skin cancers (NMSCs). Melanomas arise from the malignant transformation of melanocytes, whereas NMSCs—exemplified by basal cell carcinoma (BCC) and squamous cell carcinoma (SCC)—originate from keratinocytes and their precursor cells [282]. BCC is the most diagnosed form of skin cancer, with SCC following as the second most prevalent. Nonetheless, despite its lower incidence, advanced-stage melanoma is distinguished by its high aggressiveness, unfavorable prognosis, and is responsible for skin cancer-related deaths [283,284].

In melanoma, the PI3K (phosphoinositide 3-kinase)/PTEN/AKT/mTOR signaling pathway, which plays a crucial role in cellular homeostasis, represents the second most frequently activated pathway in disease progression, after the MAPK signaling pathway [285,286]. Additionally, various genetic mutations contribute to the overexpression of proteins that facilitate tumor invasion and infiltration into surrounding tissues, including MMPs [287]. Among the latter, MMP-2 and MMP-9 are of particular significance, as their expression is regulated through the inhibition of the NF-κB signaling pathway [288,289].

A culture-based microbial analysis of patients with melanoma identified a significant association between the genus *Corynebacterium* and advanced-stage melanoma (stage III/IV) compared to early-stage cases (stage I/II) [290]. Patients testing positive for *Corynebacterium* exhibited also a higher presence of interleukin (IL)-17-positive cells than those without detectable *Corynebacterium* colonization [290]. IL-17 is known to promote melanoma progression by upregulating IL-6 and activating the signal transducer and activator of transcription 3 (STAT3) pathway, contributing to tumor growth and immune modulation [291]. In another study around epithelial ovarian cancer, it was suggested that IL-6 stimulates MMP-9 production through phosphorylation and activation of STAT3 (p-STAT3) [292]. These findings suggest a potential mechanistic link between *Corynebacterium*-associated IL-17 upregulation and melanoma progression, highlighting the role of microbial influence on tumor-promoting inflammatory pathways and subsequent MMP-9 upregulation.

While the skin microbiome in melanoma patients remains underexplored, studies using porcine models have identified significant microbial differences between diseased and healthy skin. The melanoma-associated microbiota is characterized by an increased abundance of *Fusobacterium*, *Trueperella*, *Staphylococcus*, *Streptococcus*, and *Bacteroides* species [293,294]. Additionally, melanoma progression from in situ to invasive stages is linked to reduced gut microbiota diversity and notable alterations in microbial composition, particularly an enrichment of butyrate-producing *Clostridiales* species [295]. Butyrate promotes melanoma invasion by upregulating annexin A1 (ANXA1) and activating the EMT [296]. Furthermore, ANXA1 has been shown to facilitate tumor invasion in breast cancer through NF-κB activation and MMP-9 expression [297]. Given that ANXA1 is overexpressed in melanoma cell lines and patient samples [298], it is plausible that NF-κB may similarly contribute to increased MMP-9 expression in melanoma, paralleling its role in breast cancer progression.

SCFAs produced by both the gut microbiome and beneficial bacteria (*S. epidermis*) may modulate MMP expression through multiple mechanisms, including GPCRs, HDAC inhibition, and metabolic pathways [257]. By binding to GPCRs expressed on skin and other cell types, butyrate influences tissue metabolism and function [258]. SCFAs act through GPCRs by attenuating the activation of NF-κB signaling pathway [299], and consecutively could also potentially decrease MMP-9 expression. Moreover, HDAC4 inhibition increased the expression of MMP-1 and decreased that of type I procollagen [300]. All this can lead to a protective role of SCFAs during melanoma pathogenesis and disease progression, highlighting the dual action of SCFAs.

### 5.2. The Fundamental Role of MMPs in the Gut–Brain Axis

#### 5.2.1. The Impact of MMPs in Neurodegenerative Diseases

In the healthy nervous system, MMPs and ADAMs regulate blood–brain barrier (BBB) integrity and control bacterial and immune (PMNs, macrophages, and lymphocytes) cell infiltration to the brain, as well as cytokine signaling. Moreover, they are involved in neuronal development, repair, and regeneration by regulating stem cell differentiation, axon elongation, and remyelination [301,302]. However, aberrant MMP activity within the central nervous system (CNS) has been linked to neuronal diseases, such as multiple sclerosis (MS), Parkinson’s disease (PD), Alzheimer’s disease (AD), glioblastoma, stroke, as well as in viral and bacterial infections of the central nervous system also involving MatrixBiome. All these diseases share a common feature of an excessive neuroinflammatory response, being initiated due to compromised BBB integrity. During neuropathogenesis, the urge to recruit immune cells to damaged sites leads to the secretion of an additional load of MMPs, especially gelatinases (mostly MMP-9), by infiltrating immune cells, vascular endothelial cells, and brain cells (neurons, microglia, and astrocytes), which in turn results in the disruption of tight junctions between cerebral vascular endothelial cells and subsequent BBB damage [303,304,305,306]. Other MMP members contribute to further increase BBB permeability [307,308]. Irrespective of the purpose, which is to facilitate immune cell entry to CNS, increased MMP (mainly MMP-1 and MMP-2) activity is associated with direct neurotoxic effects and can also promote demyelination [309]. The cleaved myelin fragments together with MMP-mediated TNF-α truncation and activation, can lead to excessive inflammatory response [310].

MMP-9 is the most prevalent MMP member, ubiquitously expressed and released in brain and cerebrospinal fluid (CSF) of patients suffering from ischemic stroke, vascular dementia, AD, dolichoectasia, viral-associated neurocognitive disorders, neuro-Behcet’s disease and MS [311,312,313,314]. Moreover, MMP-9 polymorphisms are correlated with enhanced susceptibility to ischemic cerebrovascular pathologies [315]. For many of these diseases, treatment options utilizing MMP inhibitors have been assessed and reviewed, while it is interesting to note that the MS treatment IFN-β functions to inhibit T-cell-induced MMP-9 production and the associated ability of T-cells to migrate through collagen matrices [316,317]. Further, glioblastoma employs an autonomous system of MMP production and activation that facilitates glioma cell migration and invasion, implicating the production of MMP-2, MT-MMPs, and MMP-9 and subsequent activation by the serine protease plasmin [318,319,320]. Thus, to conclude, MMPs drive the course of BBB breakdown and the subsequent neuroinflammatory events that lead to the development of the acute phases of the afore-mentioned diseases.

#### 5.2.2. The Impact of MMPs: Gut–Brain Axis Interplay in Neurodegenerative Diseases

The gut–brain axis implicates communication between the two organs via metabolites, hormones, and immunological elements. Hepatic afferent nerves sense perturbations of gut microbiota and modulate the intra-intestinal immune responses, shaping gut microflora and therefore, the liver has been gaining extra attention over the past few years as a complementary component of the axis [321]. It has been shown that gut microbiota regulates microglial maturation and function, as well as neurotransmitter synthesis and release [322,323]. Microbial metabolites produced from gut microbiota are involved in gastrointestinal motility, enforce the brain and systemic innate immune response, and modulate neurodevelopmental disorders [324,325,326]. Gut microbiota dysbiosis is in turn linked to the development of various neurodegenerative diseases, such as AD and Parkinson’s [327,328]. Specifically, AD patients and mouse models exhibit shifts over proinflammatory microbiota populations (*e.g.*, enterotype III, *Escherichia*/*Shigella*), rather than ones that are linked to reduced inflammatory states (*e.g.*, enterotype I, *Firmicutes*) [329,330]. A similar pattern was observed in MS cohorts, whereas microbiota dysbiosis was linked to inflammatory taxa domination and butyrate/acetate-species producing reduction and similar observations were made for PD patients [331,332].

Microbiota dysbiosis may affect the CNS in different ways. First, by direct infection of CNS with microbes that have entered the circulation following epithelial barrier breaches or via translocation across the vagal nerve. For example, *Borrelia burgdorferi* and *Porphyromonas gingivalis* have been observed in the brain tissues of patients with AD; however, they are considered as secondary to disease implications and not the driving cause [333,334]. Alternatively, microbiota released metabolites, such as SCFAs, can directly affect microglia and astrocytes [335,336]. Finally, proinflammatory microbiota predominance in the intestines can trigger systemic inflammation, whereas the proinflammatory cytokines can activate effector T-cells that can cross the BBB and initiate neuroinflammation [337].

As far as microbiota alteration-induced regulation of MMPs in CNS is concerned, the information found in the literature is scarce, with most covering the aspects of stroke. In stroke, it seems that there is a bidirectional relationship between gut microbiota and neuropathogenesis; that is, stroke can induce gut microbiota dysbiosis via MMP-7-mediated gut barrier damage, incident to which post-stroke peripheral organ infection and increases in lethality rates are attributed. The reasons underlying this effect have been reviewed, and include post-stroke glucocorticoid-mediated immunodepression, proinflammatory cytokine-mediated enhancement of gut permeability (loss of junction-forming proteins), and gut sympathetic nerve overstimulation [338].

Infectious neuroinflammatory diseases are also characterized by increased MMP secretion in neuronal areas where the BBB has been breached. Viral infections of the CNS are associated with the macrophage-induced production of MMP-2, MMP-7, and MMP-9, while LPS can directly induce MMP-9 expression in rat astrocytes, in relevance to neuroinflammatory states [73,339]. Thus, it is supported that MMP expression and activity in the CNS is regulated in response to gut microbiota perturbations. Accordingly, acetate and butyrate have been shown to exert anti-inflammatory effects on microglial cells via inhibition of the HDAC1 and ERK/JNK/NF-κB signaling pathways, thus possibly diminishing ΜMP expression, as it is controlled by these pathways [340,341]. A combined formulation of these metabolites is undergoing clinical trials for evaluation as a treatment option for AD. Further, butyrate, as well as fecal butyrate-producing bacteria, has been shown to reduce the activity of MMP-9 in a diabetic mouse model of ischemic stroke [69]. In a similar model, plasma MMP-9 levels were found to be negatively correlated with the relative abundance of the anti-inflammatory taxa *Holdemania* and *Collinsella* in the gut and positively correlated with the proinflammatory IL-1β/IL-17/TNFα-associated *Llobaculum*, *Erysipelotrichi*, *Erysipelotrichales*, and *Erysipelotrichaceae* taxa [342].

**Figure 4:**
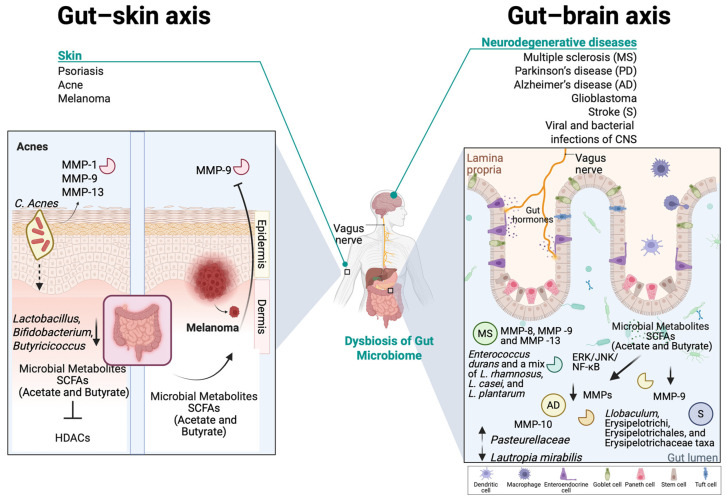
A schematic representation of the microbiota-induced expresssion of MMPs in the gut–brain axis and the gut–skin axis. On the left-hand side in the gut–skin axis and specifically in acne, *C. acnes* seem to promote the upregulation of MMPs, including MMP-1, MMP-9, and MMP-13. Simultaneously in the gut, bacteria such as *Lactobacillus*, *Bifidobacterium*, and *Butyricicoccus* produce SCFAs which protect the gut and skin barriers by inhibiting HDACs. In melanoma, gut-derived SCFAs seem to mitigate MMP-9 expression in the skin through inhibition of HDACs and butyrate-GPCR-mediated NF-κB attenuation. On the right-hand side in the gut–brain axis, MMP-10 levels are altered in AD, which is correlated to increased colonization of *Pasteurellaceae* and decreased colonization of *Lautropia mirabilis* in the gut. In MS, researchers have observed increased secretory levels of MMP-8, MMP-9, and MMP-13, and the administration of probiotic strains *Enterococcus durans* and a mix of *L. rhamnosus*, *L. casei*, and *L. plantarum* downregulated the expression of MMP-9 in the brain and CSF. In the CNS, acetate and butyrate have been shown to exert anti-inflammatory effects on microglial cells via the inhibition of HDAC1 and ERK/JNK/NF-κB signaling pathway, thus possibly diminishing ΜMP expression. In stroke, plasma MMP-9 levels were found to be correlated with the proinflammatory IL-1β/IL-17/TNFα-associated *Llobaculum*, *Erysipelotrichia*, *Erysipelotrichales*, and *Erysipelotrichaceae* taxa. This figure was created with BioRender.com.

Patients with cognitive impairment, a pathology related to AD symptoms, exhibit altered levels of MMP-10 in their CSF, which was correlated to increased colonization of *Pasteurellaceae* and decreased *Lautropia mirabilis* in the gut [343]. Human and mouse PMNs exposed to gut bacterial extracts from patients with arteriosclerotic cerebral small vessel disease demonstrated increased secretory levels of MMP-8, MMP-9, and MMP-13 [344]. In a mouse model of MS, administration of probiotic strains *Enterococcus durans* and a mix of *L. rhamnosus*, *L. casei*, and *L. plantarum* downregulated the expression of MMP-9 in the brain and CSF of the diseased animals [345] (Figure 4).

## 6. Therapies

The pathogenesis and progression of numerous inflammatory diseases and various types of cancer discussed in this review are rooted in microbiome dysbiosis and the subsequent disruption of normal MMP activity. Therefore, existing and emerging therapeutic strategies targeting the MatrixBiome in these diseases are explored.

### 6.1. Microbiome-Based Therapeutic Strategies

As discussed in this review, the human microbiome plays a crucial role in maintaining health and influencing disease, making a promising target for novel therapeutic strategies. Microbiome-targeted therapies encompass a range of approaches, such as probiotics, prebiotics, and postbiotics, bacteriotherapy, and fecal microbiota transplantation, whose goal is to restore microbial balance and improve overall health [346].

#### 6.1.1. Pro-, Pre-, and Post-Biotics-Based Therapies in MMP–Microbiome Imbalance Diseases

Probiotics, prebiotics, and postbiotics are components of microbiome-targeted therapies that aim to modulate gut health, with probiotics introducing beneficial live microorganisms, prebiotics promoting the growth of these beneficial microbes, and postbiotics providing metabolic byproducts of microbial activity that can exert health benefits [347]. Commonly recognized probiotic microorganisms include bacterial genera such as *Lactobacillus*, *Bifidobacterium*, *Enterococcus*, and *Streptococcus*, along with the yeast *Saccharomyces cerevisiae* [348]. Prebiotic compounds primarily consist of carbohydrate with diverse molecular structures that are naturally present in human dietary sources (*e.g.*, whole grains, onions, garlic, and bananas). Notable types of prebiotics include inulin, fructooligosaccharides, galactooligosaccharides, and lactulose, known to resist digestion and reach the colon where fermentation takes place thanks to the gut microbiota [349]. Postbiotics are typically all the metabolic byproducts from probiotic bacteria, such as inactivated microbial cells, cell wall components, functional proteins, peptides, SCFAs, polyamines, vitamins, bacteriocins, and other bioactive metabolites [350].

In periodontitis, oral administration of *Lactobacillus rhamnosus* SP1 as an adjunct to non-surgical therapy yielded clinical improvements comparable to scaling and root planing alone [351]. Data from multiple trials indicate that probiotics in mouthwashes effectively reduce oral pathogens, gingival index, and plaque index scores, with co-administration of chlorhexidine and fluoride demonstrating optimal efficacy against cariogenic bacteria and periodontal disease [352]. Moreover, the *Lactobacillus plantarum Y33* strain, isolated from dairy products, demonstrates significant cytotoxicity against OSCC cell lines, highlighting its potential as an adjuvant in oral cancer therapy [353]. In IBD, the probiotic strains *Limosilactobacillus fermentum* KBL374 and KBL375 seem to downregulate inflammatory cytokines and leukocyte infiltration, while also strengthening the gut barrier function by partly restoring the gut microbiota in DSS mice [354]. A recent study (NCT04223479) of UC patients demonstrated that probiotic capsules containing nine *Lactobacillus* and five *Bifidobacterium* strains promoted remission, significantly reducing C-reactive protein levels and increasing hemoglobin, hematocrit, and RBC counts, while also decreasing IgA and increasing IL-10 compared to the placebo group [355]. In CRC, the probiotic strain *Lactobacillus plantarum* YYC-3 modulated the microenvironment of colon tissue, downregulated inflammation and inhibited metastasis by downregulating VEGFA, MMP2, and MMP9 [356]. In melanoma, *Lactobacillus reuteri* stimulates cytotoxic CD8+ T-cells by secreting indole-3-aldehyde (I3A). When mice were fed a tryptophan-rich diet, which *L. reuteri* metabolized into I3A, augmented immune checkpoint inhibitor efficacy, reductions in melanoma size and improved survival were noted [357]. In psoriasis patients who were receiving local anti-psoriatic treatment, prebiotic and probiotic (*Bacillus* spp.) supplementation further alleviated symptoms by modulating cytokine activity, reducing TNFα, IL-6, and IFN-γ levels, and increasing IL-10, promoting an anti-inflammatory response [358]. Oral probiotic supplementation, particularly with Bacillus strains and *Lactobacillus rhamnosus* SP1, has been shown to improve acne by reducing lesion severity, regulating sebum excretion, and modulating gene expression related to insulin signaling, leading to enhanced clinical outcomes [359,360].

#### 6.1.2. Fecal Microbiota Transplantation—Approaches in MMP–Microbiome Imbalance Diseases

Fecal microbiota transplantation (FMT) is a therapeutic approach designed to restore gut microbiota diversity, particularly in individuals whose microbiome has been depleted due to repeated antibiotic treatments, leading to *Clostridioides* difficile infection (CDI). By transferring pre-screened donor stool into the patient’s gastrointestinal tract, FMT aims to reintroduce beneficial microorganisms, re-establish microbial balance, and improve gut functionality, making it an effective strategy for managing dysbiosis-related diseases [361]. In UC mice models exhibiting a type of IBD, FMT alleviated UC symptoms by improving DAI scores, restoring gut microbiota function, increasing SCFA levels, and inhibiting NF-κB signaling, thereby reducing inflammation [362]. FMT remains an experimental therapy for IBD due to its inconsistent efficacy, higher risk of adverse effects, and unknown long-term outcomes, as IBD patients are more prone to complications such as fever, elevated inflammatory markers, and potential bloodstream infections compared to those treated for CDI [363,364,365]. A 2017 study demonstrated that FMTs from wild mice to laboratory mice enhanced host fitness and conferred resistance to DSS/azoxymethane (AOM)-induced colorectal tumorigenesis [366]. Despite the promising in vivo model studies, a lot of risks are emerging from clinical studies regarding the possible transmission of unrecognized pathogens and the potential risk of disease-causing genes [367]. To date, no studies have investigated the impact of FMT on oral diseases; however, evidence from OSCC model mice suggests that FMT may counteract antibiotic-induced OSCC progression [368]. A case study suggests that FMT may serve as a potential therapeutic approach for severe psoriasis, as a patient receiving two FMT treatments showed significant improvements in PASI, BSA, and DLQI scores, as well as intestinal symptoms, with a marked reduction in TNF-α levels and no reported adverse effects; however, further clinical trials are required to establish its efficacy [369]. A clinical trial (NCT03341143) demonstrated that FMT combined with anti-PD-1 therapy was well tolerated and provided clinical benefits in a subset of PD-1-refractory melanoma patients by modulating the gut microbiome, enhancing CD8+ T cell activation, and altering the tumor microenvironment to overcome therapy resistance [370]. While FMT holds potential for alleviating acne symptoms, its therapeutic application remains under investigation, with no clinical trials currently dedicated to this condition. Notably, an unrelated FMT study identified *Fusicatenibacter* as a producer of butyric and valeric acids, which were associated with decreased IL-8 expression—mechanisms that may contribute to reducing inflammation in acne patients [371].

### 6.2. MMP-Based Therapeutic Strategies

Matrix metalloproteinase inhibitors (MMPIs) have been explored since the late 1990s as potential therapeutic agents for diseases characterized by excessive MMP activity, including cancer, and tissue remodeling disorders. The pharmaceutical industry has since focused on developing selective MMPIs that act specifically under pathological conditions. However, challenges remain, as many synthetic inhibitors lack specificity, exhibit poor pharmacokinetics, and inadvertently affect closely related enzymes due to the highly conserved catalytic domain shared by MMPs. Additionally, systemically administered MMPIs have demonstrated significant toxicity, limiting their clinical applications [372].

Chlorhexidine (CHX) is a biguanide antiseptic widely utilized in dentistry that portrays a pivotal role in plaque control and the reduction of gingival inflammation [373]. Beyond its antimicrobial properties, CHX has been extensively studied for its matrix MMP inhibitory effects, primarily through the chelation of calcium and zinc ions, which suppresses the activity of MMP-2, MMP-8, and MMP-9 [373,374]. This inhibition mitigates collagen degradation, thereby contributing to the preservation of dentin bond strength. Furthermore, CHX has been shown to dose-dependently inhibit the collagenolytic activity of MMP-8 released by phorbol myristate acetate-stimulated human polymorphonuclear leukocytes [374]. Despite its therapeutic potential, the high water solubility of CHX may facilitate its leaching from treated surfaces, potentially compromising its long-term efficacy in dental applications, such as periodontitis and caries [375]. To date, there are no reported associations between chlorhexidine and OSCC. However, given the widespread use of chlorhexidine in dentistry, including its presence in over-the-counter products, there are significant concerns regarding the potential development of bacterial resistance to CHX and the emergence of cross-resistance to antibiotics [376].

On a related note, Periostat, a Food and Drug Administration-approved sub-antimicrobial dose of doxycycline, inhibits MMPs (IC_50_: 2–50 μM) and enhances periodontal therapy by chelating Zn^2+^ and Ca^2+^, inhibiting latent MMP activation, and increasing TIMP-1 expression [377,378]. As a tetracycline derivative, it improves bone healing by modulating Wnt signaling while reducing osteoclast activity and promoting fibroblast collagen production, making it effective for periodontal disease without the side effects of traditional antibiotics [378]. Furthermore, doxycycline’s inhibition of MMP activity in OSCC has been associated with the suppression of tumor progression in both in vitro and in vivo mouse models, and its efficacy is currently being evaluated in a Phase 2 clinical trial (NCT03076281) in patients [379].

In CRC, Marimastat targets MMP-1, MMP-3, MMP-7, and MMP-9, reducing carcinoembryonic antigen levels in CRC; however, despite its good oral bioavailability and favorable phase III trials, it failed to improve overall survival or halt tumor progression, though combination therapies may enhance its efficacy [380]. In a different approach, miRNAs regulate MMP activity at multiple levels, with miR-34a targeting p53 to reduce MMP-1 and MMP-9, thereby limiting colon cancer proliferation and invasion [381]. Additionally, miR-29b inhibits CRC metastasis, suppresses angiogenesis, and mitigates EMT by targeting the MMP-2 gene [382]. However, targeted miRNA delivery remains challenging, with MMP-responsive PEGylated polyplexes offering a potential strategy to enhance uptake and therapeutic efficacy [383,384]. In a murine model, AB0041, an antibody with highly specific noncompetitive MMP-9 inhibitory profile, effectively reduced colon cancer xenograft growth and metastasis. Its humanized counterpart, GS-5745 (Andecaliximab or AB0041), exhibited comparable potency and selectivity, progressing to Phase I clinical trials for CD, UC, and advanced solid tumors, including CRC [385,386]. Evidence suggests that MMPs exhibit higher activity during the pre- and peri-metastatic phases of disease, indicating that restricting MMPI studies to advanced cancers may overlook critical interventional opportunities, whereas incorporating early-stage cancers in clinical trials could lead to more promising results [385].

For melanoma, the most promising results have been observed with the monoclonal inhibitory antibody DX-2400, which targets MT1-MMP and effectively slows tumor growth and metastasis in multiple in vivo mouse models of breast cancer and melanoma, both as a standalone treatment and in combination with paclitaxel or bevacizumab [387,388]. Finally, miR-143 acts as a tumor-suppressor in melanoma, where its upregulation reduces metastasis, proliferation, and enhances apoptosis in vitro [389] (Table 2).

## 7. Conclusions

The human microbiome plays a critical role in maintaining homeostasis under physiological conditions, while also influencing the onset and progression of various diseases. The intricate interplay between the microbiome, host cells, and immune cells regulates cellular communication and activates biological mechanisms that contribute to pathological conditions, particularly inflammatory diseases and cancer. The ECM serves as a pivotal mediator in this crosstalk, acting as a dynamic barrier that both microbiota and host cells must navigate to influence one another. The emerging field of MatrixBiome research explores the role of ECM in microbiome–host tissue communication.

This review highlights the role of MMPs in microbiota-driven ECM remodeling during inflammation and tumorigenesis. MMP activation can be triggered either by microbiota-derived secretory molecules, such as metabolites, or by immune cells in response to inflammatory stimuli. For example, in the OM, pathogens like *Prevotella intermedia* and *Fusobacterium nucleatum* induce the expression of MMP-1, MMP-8, and MMP-9 during periodontitis and dental caries. In OSCC, *F. nucleatum* and *Porphyromonas gingivalis* stimulate MMP-1, MMP-2, and MMP-9 expression, destabilizing adherens junctions and promoting EMT. In IBD, bacterial metabolites such as acetate, butyrate, and propionate drive the expression of MMP-1, MMP-2, MMP-3, and MMP-9, contributing to tissue damage and barrier dysfunction. Similarly, SCFAs and SBAs can disrupt gut epithelial integrity and activate the immune system through LPS-induced MMP-9 expression, which is also implicated in CRC progression.

Interestingly, the influence of the gut microbiome extends beyond the intestine, affecting distant organs via circulating microbial metabolites that modulate MMP expression. This has been observed in the gut–brain and gut–skin axes, where MMP-driven ECM remodeling contributes to the progression of neurodegenerative disorders and skin malignancies, such as melanoma. Therapeutic strategies, including the use of prebiotics, probiotics, postbiotics, and MMPIs, offer promising avenues to mitigate MMP-mediated tissue damage and disease progression. Targeting microbiome–ECM interactions could therefore provide innovative treatments for a range of inflammatory and neoplastic conditions.

## Figures and Tables

**Table 1:** The effect of microbiota secretory factors on MMP expression/activity in relation to disease.

Matrix Metalloproteinase	Metabolite/ Secretory Factor	Disease	Microbial Taxa Involved	Reference
MMP-1, MMP-3 ↓	Propionate, butyrate	IBD osteoarthritis	Not specified	[63,64]
MMP-2, MMP-9 ↓	Butyrate	IBD	Not specified	[65,66]
MMP-9 ↓	lactate	IBD	Not specified	[65]
MMP-2 ↑	TSST-1	Uteral inflammation	*Staphylococcus*	[53]
MMP-2, MMP-3, MMP-9 ↑	PLC	Gingivitis, periodontitis	*Bacillus Cereus*	[67]
MMP-7 ↑	Enterotoxin	IBD	*Bacteroides fragilis*	[52]
MMP-8, MMP-9 MMP-2,-14,-11,-17 ↑	Dentilisin	OSCC	*Treponema denticola*	[68]
MMP-9 ↓	Butyrate	Stroke	Not specified	[69]
MMP-9 and total MMP activity ↑	Candidalysin	OSCC	*Candida albicans*	[70,71]
MMP-9 ↓	SSL5	Not specified	*Staphylococcus* *aureus*	[72]
MMP-7 ↑	Flagellin	Cystic fibrosis	*Pseudomonas aeruginosa*	[51]
MMP-9 ↑	LPS	CRC, airway and neuro- inflammation	Not specified	[54,58,73]
MMP-10 ↑	DCA	OC	Not specified	[74]
MT4-MMP ↑	PLC	Not specified	Not specified	[75]
↑ upregulation	↓ downregulation			

**Table 2:** MMP-based therapeutic approaches with regard to disease.

Disease	Therapeutic Agent /Approach	Target	Result	Reference
Periodontitis	*Lactobacillus* *rhamnusus* SP1	Not specified	Comparable results to surgical approaches	[351]
	Chlorhexidine	MMP-2,-8,-9	↓ Gingival inflammation	[373]
	Doxycycline	Inhibits MMPs ↑ TIMP-1	↓ Osteoclast activity ↑ Collagen production	[377,378]
Caries	Chlorhexidine	MMP-2,-8,-9	↓ Plaque formation	[373]
OSCC	*Lactobacillus* *plantarum* Y33	Not specified	Cytotoxicity in OSCC cell lines	[351]
	FMT	Not specified	Counteracts antibiotic- induced OSCC in mice	[368]
UC	*Limosilactobacillus* *fermentum* KBL374 and KBL375	Not specified	↓ Inflammatory cytokines, leukocyte infiltration Strengthening the gut barrier	[353]
	Probiotic capsules	Not specified	↓ C-reactive protein, IgA ↑ Hemoglobin, hematocrit, RBC, IL-10	[355]
	FMT from wild mice to DSS/AOM mice	Not specified	Resistance to colorectal tumorigenesis	[365]
	Andecaliximab (GS-5745, AB0041)	MMP-9	Reduced symptoms	[386,387]
CRC	*Lactobacillus* *plantarum* Y33	Not specified	TME modulation via ↓ VEGFA, MMP-2, MMP-9 ↓ Inflammation, metastasis	[356,390]
	miR-34a	p53	↓ MMP-1, -9	[381]
	Andecaliximab (GS-5745, AB0041)	MMP-9	↓ Tumor growth and metastasis	[386,387]
Melanoma	*Lactobacillus reuteri*	I3A stimulates cytotoxic CD8+ T cells	↓ Melanoma size ↑ ICI efficacy, survival	[357]
	FMT + anti-PD-1 (NCT03341143)	↑ CD8+ T cell activation	Modulation of gut microbiome Altered TME to overcome therapy resistance	[370]
	DX-2400 (mAb)	MT1-MMP	↓ Tumor growth and metastasis	[388,389]
	*miR-143*	↓ MMP-9	↓ Metastasis, proliferation ↑ Apoptosis	[389]
Psoriasis	*Bacilus* spp.	↓ TNFα, IL-6, IFN-γ ↑ IL-10	Symptom alleviation due to the anti-inflammatory effects	[358]
	FMT	↓ TNFα	Improvement in quality of life	[375]
Acne vulgaris	*Bacillus* and *Lactobacillus rhamnosus* SP1	Not specified	Regulation of lesion severity and sebum excretion	[364,365]
	FMT with *Fusicatenibacter*	Butyric and valeric acid → ↓ IL-8	↓ Inflammation	[371]
↑ upregulation ↓ downregulation				

## Abbreviations

The following abbreviations are used in this manuscript:

AD	Alzheimer’s disease
ADAMs	Disintegrin and metalloproteinases
ADAMTSs	Disintegrin and metalloproteinases with thrombospondin motifs
AMPs	Antimicrobial peptides
ANXA1	Annexin A1
BAs	Bile acids
BCC	Βasal cell carcinoma
BSH	Bile salt hydrolase
CD	Crohn’s disease
CDI	Clostridioides difficile infection
CHX	Chlorhexidine
CRC	Colorectal cancer
CSF	Cerebrospinal fluid
DCA	Deoxycholic acid
DSS	Dextran sodium sulfate
ECM	Extracellular matrix
EMT	Epithelial-to-mesenchymal transition
FDA	Food and Drug Administration
FMT	Fecal microbiota transplantation
GPCRs	G protein-coupled receptors
GTFs	Glucosyltransferases
HDACs	Histone deacetylases
IBD	Inflammatory bowel disease
IL	Interleukin
LCA	Lithocholic acid
LPS	Lipopolysaccharide
MAPK	Mitogen-activated protein kinase
MMPs	Matrix metalloproteinases
NMSCs	Non-melanoma skin cancers
MS	Multiple sclerosis
OC	Esophageal cancer
OM	Oral microbiome
OSCC	Oral squamous cell carcinoma
PD	Parkinson’s disease
PLC	Phospholipase C
RANKL	Receptor activator of NF-kB ligand
SCC	Squamous cell carcinoma
SCFAs	Short-chain fatty acids
TF	Transcription factor
TLR	Toll-like receptor
UC	Ulcerative colitis
uPAR	Urokinase receptor
